# La-Doped Sm_2_Zr_2_O_7_/PU-Coated Leather Composites with Enhanced Mechanical Properties and Highly Efficient Photocatalytic Performance

**DOI:** 10.3390/ma17071575

**Published:** 2024-03-29

**Authors:** Liliang Chen, Weiguo Li, Xianbo Hou, Gang Feng

**Affiliations:** 1 College of Aerospace Engineering, Chongqing University, Chongqing 400030, China; chenliliang198601@163.com (L.C.); xianbo_hou@126.com (X.H.); 2 Chongqing Changan Global R&D Center, Changan Automobile Co., Ltd., Chongqing 400023, China; fengg@changan.com.cn

**Keywords:** La doping, Sm_2_Zr_2_O_7_, finished leather, photocatalysis, mechanical properties

## Abstract

Flexible La-doped Sm_2_Zr_2_O_7_/polyurethane (PU) coated leather composites were synthesized using a one-step hydrothermal method, with highly efficient photocatalytic degradation properties by coating the La-doped Sm_2_Zr_2_O_7_/PU emulsion onto the leather and drying it. The phase composition and optical properties of the as-prepared photocatalytic material were systematically characterized. The result revealed that La was doped in Sm_2_Zr_2_O_7_ successfully, and the prepared samples still possessed pyrochlore structure. The absorption edge of the prepared samples exhibited a red-shift with the increase in La doping, indicating that La doping could broaden the absorbance range of the La-doped Sm_2_Zr_2_O_7_ materials. The catalytic performance of La-doped Sm_2_Zr_2_O_7_/PU composite emulsion coating on the photocatalytic performance of leather was studied with Congo red solution as the target pollutant. The results showed that the best photocatalytic property was found in the 5% La-doped Sm_2_Zr_2_O_7_ nanomaterial at a concentration of 3 g/L. The resulting 5% La-doped Sm_2_Zr_2_O_7_ nanomaterial exhibited a high specific surface area of 73.5 m^2^/g. After 40 min of irradiation by a 450 W xenon lamp, the degradation rate of Congo red reached 93%. Moreover, after surface coating, the La-doped Sm_2_Zr_2_O_7_/PU coated leather composites showed obviously improved mechanical properties, as the tensile strength of La-doped Sm_2_Zr_2_O_7_/PU coated leather composites increased from 6.3 to 8.4 MPa. The as-prepared La-doped Sm_2_Zr_2_O_7_/PU coated leather composites with enhanced mechanical properties and highly efficient photocatalytic performance hold promising applications in the treatment of indoor volatile organic compounds.

## 1. Introduction

The main source of indoor pollutants is volatile organic compounds, which mainly contain toxic and harmful substances such as aldehydes and benzene series. When working or studying in this environment for a long time, the human body is prone to fatigue, low mood, and even dizziness and vomiting. Therefore, the treatment of volatile organic compounds within indoor spaces is crucial [1,2]. Photocatalytic degradation technology has the advantages of mild reaction conditions and green environmental protection and is considered a treatment method for volatile organic compounds with wide application prospects [3,4,5].

Leather coating materials refer to the formation of a layer or several layers of film onto the surfaces of leather so as to improve the appearance of the leather, increase its functional characteristics, and expand its scope of use. In order to prevent people from being in a polluted indoor environment for a long time, it is of great significance to develop a leather finishing material that can photocatalyze the degradation of volatile organic compounds. PU coatings are widely used for leather coating because of their advantages of low price, soft feel after film formation, and high light transmittance. However, their defects, such as low added value and poor mechanical properties, limit their further application [6,7].

Oxide semiconductor materials, such as TiO_2_, ZnO, Bi_2_WO_6_, and La_2_Zr_2_O_7_ [8,9,10,11,12], are widely used in environmental treatment due to their unique physical and chemical properties and their advantages of a stable structure, low cost of large-scale production, and environmental protection. Among them, Sm_2_Zr_2_O_7_ is a composite oxide with a layered three-dimensional pyrochlore structure [13,14]. The bond angle of Zr-O-Zr within this crystal structure is close to 180°. This unique crystal structure contributes to the movement of photogenerated carriers, giving Sm_2_Zr_2_O_7_ its good photocatalytic activity [15]. However, standalone Sm_2_Zr_2_O_7_ has limited photoresponse range and a high recombination rate of photogenerated electrons and holes [16], which limits its application in the field of photocatalysis. Researchers have found that the use of rare earth ion doping in Sm_2_Zr_2_O_7_ can effectively expand the visible spectral response range, enhance light absorption capacity, and thus significantly improve photocatalytic activity. Therefore, rare earth elements have received more and more attention in the modification of photocatalysts [17,18,19,20]. Although it has been reported in the literature that rare earth-doped Sm_2_Zr_2_O_7_ has been prepared by the stearic acid method, and it has been found that rare earth-doped ions can promote photocatalytic performance [21,22], the preparation process for Sm_2_Zr_2_O_7_ requires high-temperature sintering (900 °C) and long reaction times. Additionally, the mechanism by which rare earth ion doping promotes photocatalytic activity is not very clear.

In this study, we prepared La-doped Sm_2_Zr_2_O_7_ photocatalyst using a one-step hydrothermal method at low temperature. The La-doped Sm_2_Zr_2_O_7_ photocatalyst was introduced into polyurethane emulsion to prepare La-doped Sm_2_Zr_2_O_7_/PU coated leather composites, which could effectively degrade indoor pollutants and was essential for the treatment of volatile organic compounds in indoor spaces. The effect of La doping on the catalytic performance of Sm_2_Zr_2_O_7_ in the photocatalytic degradation of Congo red and the effect of La-doped Sm_2_Zr_2_O_7_/PU on the mechanical properties of leather composites were studied, and the reasons behind La-doping photocatalytic activity were analyzed.

## 2. Materials and Methods

### 2.1. Raw Materials

ZrOCl_2_·8H_2_O with industrial purity was purchased from Zibo Huantuo Chemical Co., Ltd., Zibo, China; samarium Sm(NO_3_)_3_ nitrate with analytical purity was purchased from Guangdong Rier Chemical Technology Co., Ltd., Guangzhou, China; lanthanum nitrate La(NO_3_)_3_ with analytical purity was purchased from Guangdong Rier Chemical Technology Co., Ltd.; ammonia, sodium hydroxide, and anhydrous ethanol water, all with analytical purity, were purchased from Beijing Chemical Plant, Beijing, China; self-made deionized water was used for the experiment. Polyurethane emulsion with analytical purity was purchased from Shanghai Aladdin Biochemical Technology Co., Ltd., Shanghai, China, and PU leather was purchased from Shanghai Huafeng Microfiber Technology Co., Ltd., Shanghai, China.

### 2.2. Preparation of La-Doped Sm_2_Zr_2_O_7_/PU Coated Leather Composites

Sm(NO_3_)_3_ and ZrOCl_2_·8H_2_O were weighed, respectively, according to the amount of substance n (Sm):n (Zr) = 1:1, and dissolved in 50 mL of deionized water in turn. Under the condition of magnetic stirring, a certain amount of ammonia was added to adjust the pH value of the solution within the range of 5.5–9.0, and the white mixed precipitate was obtained by centrifugation. Finally, the mineralizer 11 M KOH solution was poured into the sediment to form a slurry. After thorough stirring, the whole solution was transferred to a Teflon-lined hydrothermal kettle. The kettle was sealed and placed in a constant temperature oven to react at 190 °C for 24 h. After the reaction, the product was naturally cooled to room temperature, washed with water five times, washed with ethanol two times, centrifuged, and dried at 70 °C for 4 h to obtain the product. La-doped Sm_2_Zr_2_O_7_ is prepared by adding La(NO_3_)_3_ to the Sm(NO_3_)_3_ solution, as the mass fraction of La is 1% (mass fraction), 3%, 5%, and 7%, which were named as x% La-doped Sm_2_Zr_2_O_7_ (x = 1, 3, 5, and 7), respectively.

La-doped Sm_2_Zr_2_O_7_ was evenly dispersed in PU emulsion under stirring and ultrasonic conditions, and the composite emulsion was coated on the leather; the coating amount was about 250–350 g/m^2^. After each spray was finished, it was dried in the oven at 65 °C. After spraying, rolling, and embossing, the leather composites could be prepared.

### 2.3. Characterization of Product Properties

The phase composition of a photocatalyst sample was tested using an X-ray diffraction (XRD) analyzer (PANalytical, Almelo, The Netherlands), and structural analysis data were obtained. The size and morphology of photocatalyst samples were analyzed by field emission transmission electron microscopy (TEM) from FEI Company, Dreieich, Germany. The specific surface area of photocatalytic samples was measured at −196 °C with an ASAP2020 nitrogen adsorption instrument (Norcross, GA, USA). The light absorption performance of the photocatalyst was tested using a Hitachi U-3310 UV-visible photometer (Tokyo, Japan). The mechanical properties of finished leather were tested using a C43 universal material testing machine made by MTS Systems. A fully automatic fabric breathability tester (Wenzhou Jigao Testing Instrument Co., Ltd., Wenzhou, China) was used to test and analyze the breathability of finished leather, according to GB/T5453-1997 [23].

### 2.4. Evaluation of Photocatalytic Performance

In this study, Congo red dye was used as the target degradation of simulated organic matter rather than real-time degradation, for the following reasons: 1. Congo red is a representative azo dye, possessing chemical structures (such as benzene ring, naphthalene ring) that are difficult to degrade; 2. commercial dyes have little harm to the human body and are easy to use in experiments.

The leather sample (1 cm × 5 cm) treated with the photocatalyst was put into a test tube containing an 80 mg/L Congo red aqueous solution. A xenon lamp with 450 W was used as the simulated light source for the photocatalytic degradation reaction. An absorbance test of the extracted solution was carried out using an ultraviolet spectrophotometer every 10 min, and the concentration change of the Congo red solution was analyzed through absorbance measurement, according to the Lambert–Beer law. Dark experiments (without irradiation), blank experiments (in the absence of La-doped Sm_2_Zr_2_O_7_/PU), La-doped Sm_2_Zr_2_O_7_/PU under irradiation, and a contrast test of Degussa P25 TiO_2_ under irradiation were conducted, respectively.

The degradation rate of Congo red was calculated by:

(1) $$\gamma =\frac{A_{0}-A_{t}}{A_{0}}\times 100\%,$$

where *γ* is the degradation rate; *A*_0_ and *A_t_* are the initial absorbance values of the sample solution and the absorbance values during the degradation of *t*, respectively.

### 2.5. Mechanical Property Test

(1) Tensile strength and elongation at break

After the coated leather samples were placed in a constant temperature and humidity box for 24 h, on average, three samples (the shape of the sample was a dumbbell) were cut out, and the tensile strength and elongation at break of the film were measured using a tensile testing machine (the tensile rate was 100 mm/min).

The tensile strength is calculated by:

(2) P=F/S,

where P is the tensile strength of the sample, N/mm^2^; F is the force on the fracture section of the specimen when it breaks, N; S is the area of the specimen fracture surface, mm^2^.

The elongation at break can be obtained by calculating:

(3) $$E=\frac{L_{1}-L_{0}}{L_{0}}\times 100\%,$$

where E is the elongation at break, 100%; *L*_1_ is the length of the stressed part of the specimen at fracture, mm; *L*_0_ is the original sample length, mm.

(2) Breathability

After the finished leather sample was air-conditioned in a constant temperature and humidity box for 24 h, a round sample with a diameter of 5.5 cm was cut. The permeability of leather samples was tested using a leather permeability tester. Each sample was measured in parallel more than twice, and the error between parallel experiments was less than 0.5 s. The calculation formula is as follows:

(4) $$K=100\times 3600/S(t-t_{0}),$$

where *K* is the sample permeability, mL/(cm·h); *t* is the time required for a specified area sample to pass 100 mL of air, s; *t*_0_ is the time required for the blank test, s; *S* is the specimen area through air, cm^2^.

## 3. Results and Discussion

**Figure 1:**
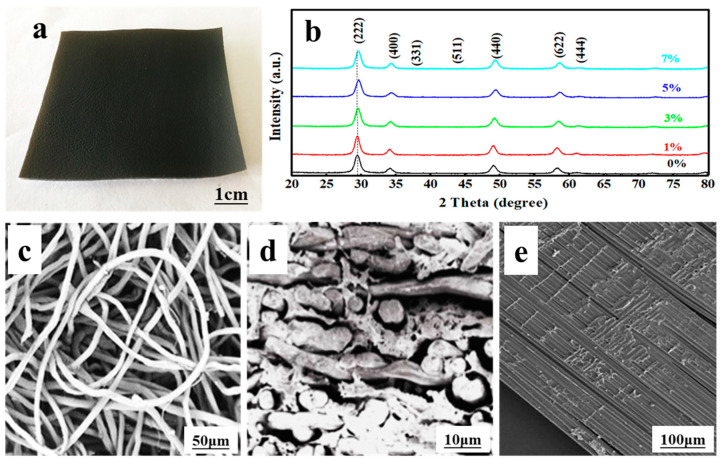
(**a**) Photograph of the flexible La-doped Sm_2_Zr_2_O_7_/PU coated leather composites; (**b**) XRD patterns of Sm_2_Zr_2_O_7_ photocatalysts with various doping contents of La; (**c**) the leather before coated La-doped Sm_2_Zr_2_O_7_/PU; (**d**) the leather after coated La-doped Sm_2_Zr_2_O_7_/PU; (**e**) the surface morphology of La-doped Sm_2_Zr_2_O_7_/PU coated leather composites.

The photograph of the flexible composite is shown in Figure 1a, where it can be seen that the La-doped Sm_2_Zr_2_O_7_/PU has been coated onto the surface of the composite. It can be seen from Figure 1b that the addition of La has no significant effect on the structure of the Sm_2_Zr_2_O_7_ crystal. Compared with the standard XRD pattern, it can be seen that each curve in the figure corresponds to the cubic crystal system of Sm_2_Zr_2_O_7_ (JCPDS card No. 24-1012) with a pyrochlore structure. The size of Sm_2_Zr_2_O_7_ particles is estimated to be about 15 nm using Scherrer’s formula. However, with the partial substitution of Sm^3+^ ions by La^3+^ ions, the XRD pattern of La-doped Sm_2_Zr_2_O_7_ is slightly offset compared with pure Sm_2_Zr_2_O_7_. The diffraction peak near 29.4° shifts slightly toward a higher angle with the increase in La-doping amount. The results show that La^3+^ ions are partially substituted and enter the lattice position of Sm^3+^ ions, but the pyrochlore type (A_2_B_2_O_7_) structure remains. The slight deviation of the diffraction peak may be caused by the different ion radii of La^3+^ (1.063 Å) and Sm^3+^ (1.098 Å). Although La-doping does not change the crystal structure of the samples, the intensity of some diffraction peaks of the sample is slightly weakened, and the width is slightly increased after doping, indicating that the grain size of Sm_2_Zr_2_O_7_ is slightly decreased due to La entering the lattice. Figure 1c–e shows the microstructure of the composite material, indicating that La-doped Sm_2_Zr_2_O_7_/PU was well-immersed into the leather.

**Figure 2:**
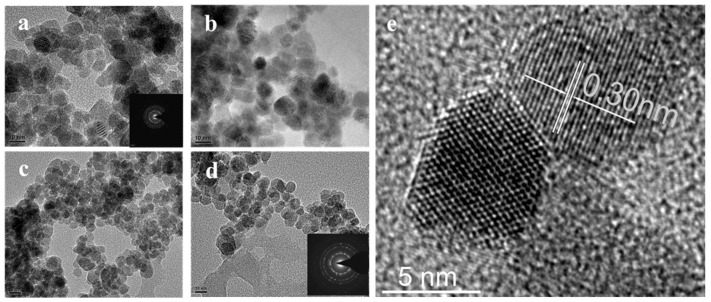
(**a**–**d**) Morphology images of Sm_2_Zr_2_O_7_ photocatalysts with various doping contents of La: (**a**): 0%; (**b**): 3%; (**c**): 5%; (**d**): 7%; (**e**) high-resolution images of particle growth.

Figure 2a–d shows the TEM morphology of La-doped Sm_2_Zr_2_O_7_ with doping amounts of 0, 3%, 5%, and 7%, respectively. The obtained samples have similar nanostructures and uniform grain size, and the grain size of the samples changes minimally with the increase in La-doping amount (~0.3 nm, as shown in Figure 2e). As can be seen from the electron diffraction graphs illustrated in Figure 2a,d, crystallinity deteriorates with the increase in La-doping amount. The reason for this may be that La-doping infiltrates into the lattice, and La^3+^ replaces Sm^3+^, resulting in defects in the lattice.

**Figure 3:**
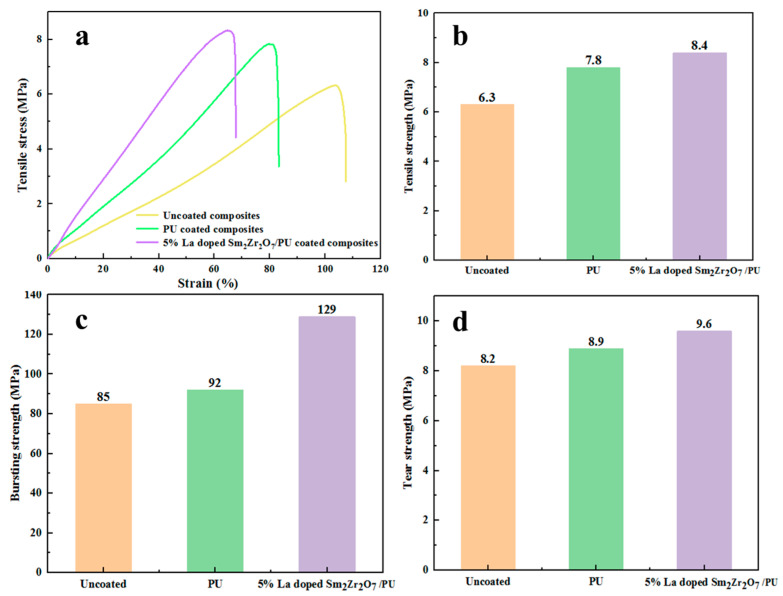
(**a**) Tensile stress–strain curves of different leather composites (uncoated composites, PU-coated composites, 5% La-doped Sm_2_Zr_2_O_7_/PU-coated composites); (**b**) tensile strength, (**c**) bursting strength, and (**d**) tear strength of different leather composites (uncoated composites, PU-coated composites, 5% La-doped Sm_2_Zr_2_O_7_/PU-coated composites).

Compared with uncoated leather and leather coated with pure PU emulsion, the tensile strength of leather coated with 5%-La-doped Sm_2_Zr_2_O_7_/PU composite emulsion is significantly improved, which may be due to the crystal structure of La-doped Sm_2_Zr_2_O_7_, and its rigid structure could enhance the tensile strength of the leather composites (Figure 3a,b). Moreover, the elongation at break of the coated leather is lower than that of uncoated leather and PU emulsion-coated leather composites. Compared with uncoated leather and pure PU emulsion-coated leather composites, the tear strength and bursting strength of the leather after composite emulsion coating are significantly improved. Compared with pure PU emulsion coating, the tear strength and disintegration strength of the leather composites after being coated with 5%-La-doped Sm_2_Zr_2_O_7_/PU emulsion are increased by 8% and 52%, respectively. This is mainly due to the fact that La-doped Sm_2_Zr_2_O_7_, as a crystalline material, has high stiffness properties, which can effectively enhance the strength of leather composites when introduced into the PU matrix.

**Figure 4:**
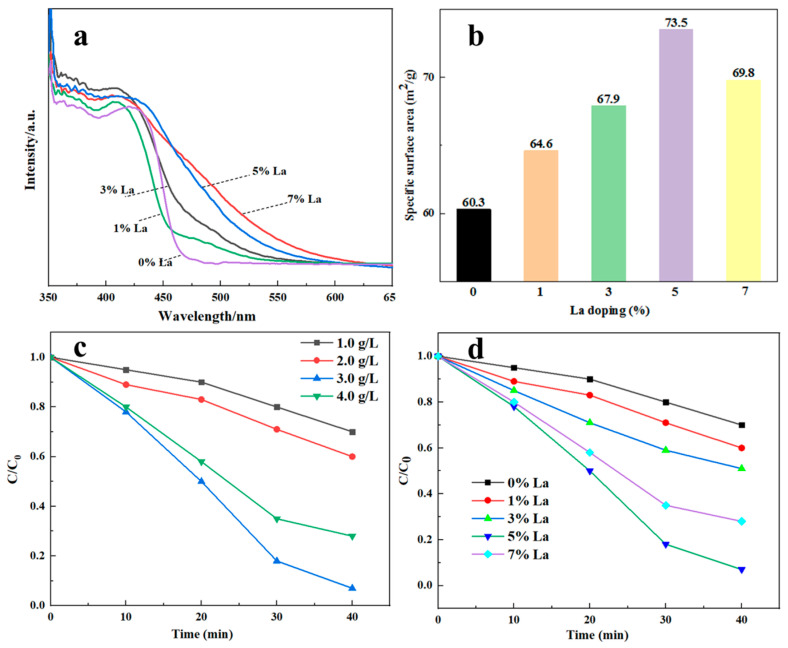
(**a**) UV-vis spectrometer of Sm_2_Zr_2_O_7_ with various doping contents of La; (**b**) specific surface area; (**c**) effect of Sm_2_Zr_2_O_7_ concentration on catalytic performance; (**d**) effects of La contents in Sm_2_Zr_2_O_7_ on Congo red degradation efficiency.

The prepared sample shows visible light absorption properties before and after La doping (Figure 4a). The resulting products produced after complete photocatalytic activity of dyes are complex and diverse [24,25,26]. The catalytic mechanism is as follows: under the condition of illumination, electrons and holes are generated in the conduction band and valence band, respectively, forming electron–hole pairs. h+ can oxidize OH^−^ and H_2_O molecules adsorbed on the surface of Sm_2_Zr_2_O_7_ to form hydroxyl radicals OH^−^. These radicals OH^−^, attached to the surface of Sm_2_Zr_2_O_7_ are strong oxidizing agents that can oxidize adjacent organic matter and diffuse into the liquid phase to oxidize organic matter. Through a series of oxidation processes, CO_2_ and H_2_O are finally oxidized, thus completing the degradation of organic matter. Compared with pure Sm_2_Zr_2_O_7_, the absorption of the doped material moved significantly in the direction of long waves, and the moving range of the absorption edge increased with the increase in La-doping amount. The absorption edges of pure Sm_2_Zr_2_O_7_ and Sm_2_Zr_2_O_7_ with La-doping levels of 1%, 3%, 5%, and 7% are 460 nm, 462 nm, 470 nm, 473 nm, and 485 nm, respectively. The absorption peak *λ_g_* of La-doped Sm_2_Zr_2_O_7_ gradually increases with the increase in doping amount, and the relationship between the semiconductor light absorption threshold *λ_g_* and the band gap width *E_g_* is as follows:

(5) $$E_{g}=1240/\lambda_{g},$$

The band gap widths of each material can be calculated as 2.70 eV, 2.68 eV, 2.64 eV, 2.62 eV, and 2.56 eV, respectively. This shows that La doping can widen the light absorption range and reduce the band gap of the material. The change in material bandgap width may be due to the fact that the 3D orbital of doped La generates impurity levels in the Sm_2_Zr_2_O_7_ bandgap, thus reducing the energy gap of Sm_2_Zr_2_O_7_.

As can be seen from specific surface area tests, the specific surface area of Sm_2_Zr_2_O_7_ without La doping is 60.3 m^2^/g, while the specific surface area of Sm_2_Zr_2_O_7_ with 1%, 3%, 5%, and 7% La-doping content is 64.6 m^2^/g, 67.39 m^2^/g, 73.5 m^2^/g, and 69.8 m^2^/g, respectively (Figure 4b). With the increase in La-doping amount, the specific surface area of the sample increases first and then decreases, reaching the maximum when the La-doping amount is 5%. It can be inferred that 5% La-doped Sm_2_Zr_2_O_7_ has higher activity. The change in specific surface area is related to the continuous increase in doping amount. As the doping number increases, the crystal structure may go through changes such as inhibited growth of grains and limited movement of grain boundaries. These changes can cause the microstructure inside the crystal to become more complex, thus increasing the specific surface area. With the continuous increase in doping amount, the crystal structure gradually tends to be stable, and the rearrangement of grains and grain boundaries may lead to a gradual reduction in specific surface area. After a certain critical point, the structure of the crystal begins to readjust, resulting in a reduced specific surface area [27].

Figure 4c shows the effect of the concentration of synthesized La-doped Sm_2_Zr_2_O_7_ on catalytic performance. It can be seen that the concentration of the catalyst has a great influence on the photocatalytic effect. The light degradation rate of Congo red increases with the increase in La-doped Sm_2_Zr_2_O_7_ concentration, and the light degradation rate of Congo red reaches its highest when the catalyst concentration is 3 g/L. As the catalyst concentration continues to increase, the degradation rate decreases instead. The reason for the reduction in degradation rate may be attributed to the high catalyst concentration, causing the light absorption capacity of the solution to be reduced due to light scattering.

With the increase in La-doping amount, the catalytic efficiency first increases and then decreases (Figure 4d). Moreover, the catalytic activity of La-doped Sm_2_Zr_2_O_7_ is the best when the doping amount of La is 5%, and the degradation rate is 93% in 40 min, while the degradation amount of pure Sm_2_Zr_2_O_7_ and 1%-La-doped Sm_2_Zr_2_O_7_ is 30.1% and 42.6%, respectively. The test results of photocatalytic performance show that La doping can significantly improve the photocatalytic performance of Sm_2_Zr_2_O_7_. The following reasons may be responsible for the enhancement of the photocatalytic activity of Sm_2_Zr_2_O_7_ due to appropriate La doping [16]. (1) It can be seen from Figure 4d that the absorption wavelength of the sample after La-doped Sm_2_Zr_2_O_7_ is redshifted. The band gap of the sample is narrowed, and the response is stronger in the visible light region with a wavelength greater than 400 nm, achieving more visible light absorption; (2) doping La^3+^ can act as effective electron acceptors to capture photogenerated electrons transitioning from the valence band to the conduction band and promoting the effective separation of photogenerated electrons and holes; (3) La doping makes the grain size of Sm_2_Zr_2_O_7_ smaller and the specific surface area larger, which may improve the adsorption ability of the catalyst to the reaction molecules. Moreover, as La^3+^ replaces Sm^3+^, the defects in the catalyst also increase, which serve as catalytic active points and increase the catalytic activity of the La-doped Sm_2_Zr_2_O_7_. However, when the doping amount of La is too large, excess La particles are deposited on the surface of Sm_2_Zr_2_O_7_, which hinders the photocatalytic reaction, accelerating the photogenerated electron and photogenerated hole recombination, thus reducing the photocatalytic activity.

The photodegradation reaction is a quasi-first-order reaction, and its kinetic reaction can be expressed as:

(6) $$ln\left(\frac{C_{0}}{C}\right)=kt$$

**Figure 5:**
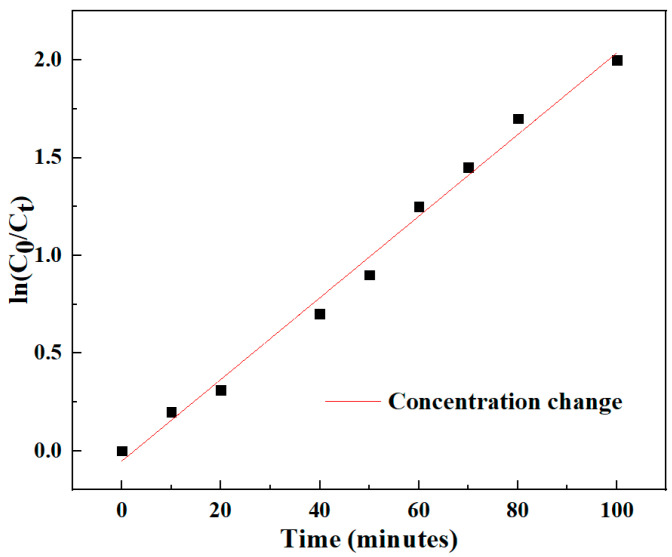
The relationship of concentration changes of Congo red solution under different light times.

*k* is the apparent reaction rate constant, *C*_0_ is the initial concentration of Congo red, and *C* is the concentration of Congo red at the reaction time *t*. Figure 5 shows the relationship between the concentration changes of Congo red solution under different illumination times. It can be seen from the figure that the reaction conforms to the quasi-first-order reaction, and ln (*C*_0_*/C*) changes in a linear relationship with time.

**Figure 6:**
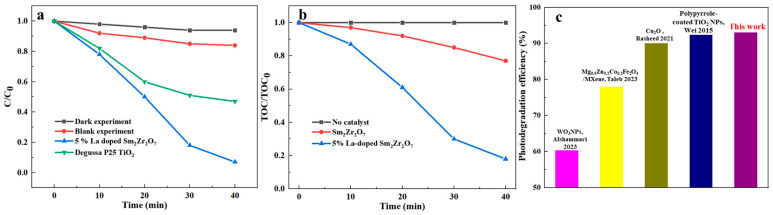
(**a**) The degradation rate (C/C_0_) of Congo red as a function of irradiation time (C_0_ and C represent the equilibrium concentration of Congo red before and after UV irradiation, respectively): a dark experiment (without irradiation); a blank experiment (in the absence of La-doped Sm_2_Zr_2_O_7_/PU); 5% La-doped Sm_2_Zr_2_O_7_/PU under irradiation; and Degussa P25 TiO_2_ under irradiation; (**b**) the TOC rate (TOC/TOC_0_) of Congo red as a function of irradiation time: a dark experiment (without irradiation); an experiment without a catalyst; Sm_2_Zr_2_O_7_/PU under irradiation; 5% La-doped Sm_2_Zr_2_O_7_/PU under irradiation; (**c**) the photocatalytic efficiency of 5% La-doped Sm_2_Zr_2_O_7_/PU on Congo red was compared with other reported photocatalysts [25,28,29,30].

Subsequently, the high activity of 5% La-doped Sm_2_Zr_2_O_7_/PU can also be confirmed from Figure 6a. In addition to experiments with La-doped Sm_2_Zr_2_O_7_/PU and irradiation, dark experiments and blank experiments were investigated in the absence of irradiation with La-doped Sm_2_Zr_2_O_7_/PU or in the presence of irradiation without La-doped Sm_2_Zr_2_O_7_/PU. Figure 6 demonstrates that almost no Congo red degradation occurs, while Figure 6 shows that only a small quantity of Congo red is degraded (less than 15%, which can be interpreted by the photolysis effect). Moreover, in order to exhibit the mineralization of organic pollution, reduction in the TOC is also presented in Figure 6b to study the complete mineralization efficiency of Congo red. For the purpose of comparison, the photocatalytic degradation of Congo red was carried out using Degussa P25 TiO_2_ and La-doped Sm_2_Zr_2_O_7_/PU under the same conditions. These experimental results showed that La-doped Sm_2_Zr_2_O_7_/PU exhibited better photocatalytic activity than Degussa P25 TiO_2_. Moreover, the photocatalytic efficiency of 5% La-doped Sm_2_Zr_2_O_7_/PU on Congo red was compared with other reported photocatalysts, as shown in Figure 6c. The results demonstrate that the as-prepared 5% La-doped Sm_2_Zr_2_O_7_/PU exhibits better catalytic performance (93%) than that of recently reported photocatalysts at nearly the same catalytic time [25,28,29,30].

**Figure 7:**
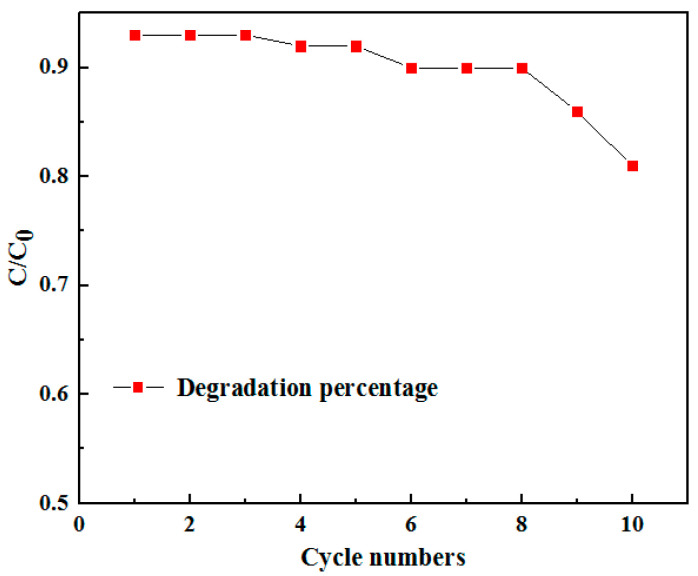
Reusable tests for La-doped Sm_2_Zr_2_O_7_/PU.

In addition to photocatalytic degradation activity, another important index to characterize photocatalysts is catalytic stability, especially for composite structure where component separation may occur. In order to evaluate the catalytic stability of the La-doped Sm_2_Zr_2_O_7_/PU composite emulsion, a cyclic stability test of the La-doped Sm_2_Zr_2_O_7_/PU composite was carried out during the photocatalytic degradation process. The analysis of experimental results is shown in Figure 7. As shown in Figure 7, the degradation rate of the La-doped Sm_2_Zr_2_O_7_/PU composite was almost unchanged after eight photocatalytic degradation cycles, indicating that the La-doped Sm_2_Zr_2_O_7_/PU composite had good cyclic stability. However, the catalytic degradation performance gradually decreased after more than eight cycles of cyclic testing. This can be attributed to the continuous adsorption of Congo red in the La-doped Sm_2_Zr_2_O_7_/PU composite coating failing to remove it in time, resulting in a gradual reduction in its photocatalytic degradation efficiency.

**Figure 8:**
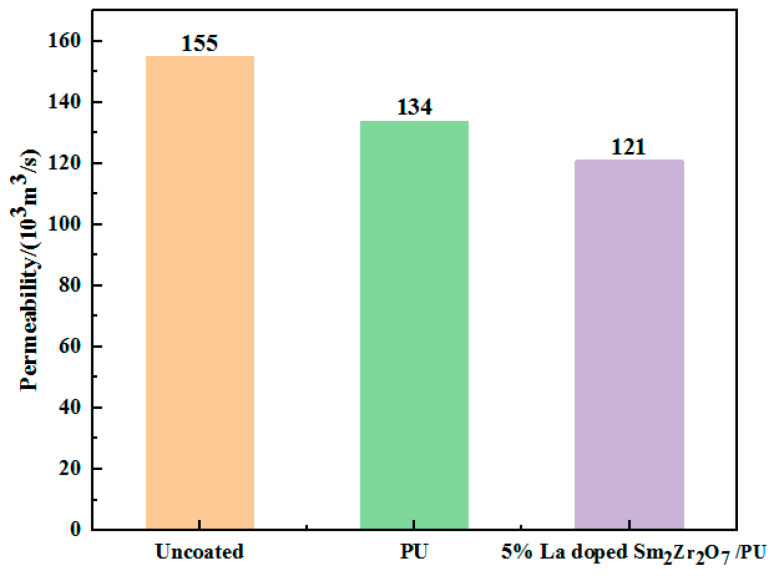
Water vapor permeability of different leather composites.

This leather material, used in the field of automotive interior or home decoration, needs to have good air permeability because air permeability allows moisture and gas to pass through, improving ride comfort, especially in hot weather or during long drives. The air permeability of leather after coating with 5%-La-doped Sm_2_Zr_2_O_7_/PU emulsion is shown in Figure 8. Compared with leather coated with pure PU emulsion, the air permeability of leather coated with emulsion is slightly decreased, which is mainly due to the formation of film on the leather surface during the coating process of the emulsion. La-doped Sm_2_Zr_2_O_7_ nanoparticles can permeate into the leather, resulting in a decrease in the permeability rate of the leather composites.

## 4. Conclusions

In this study, La-doped Sm_2_Zr_2_O_7_ was successfully synthesized by coating the La-doped Sm_2_Zr_2_O_7_/PU emulsion onto the leather and drying, which made the absorption edge redshift, decreased the band gap, and increased the light absorption ability and specific surface area. The photocatalytic rate of La-doped Sm_2_Zr_2_O_7_ composite for Congo red reached 93%, and the mechanical properties of the composite were also significantly improved after coating with La-doped Sm_2_Zr_2_O_7_/PU emulsion. The developed La-doped Sm_2_Zr_2_O_7_/PU-coated leather composites have potential application prospects for the purification treatment of indoor volatiles.

## Data Availability

Data are contained within the article.
